# Breakthrough Tuberculosis in a Large Cohort of People Living With HIV on Preventive Treatment

**DOI:** 10.1111/tmi.70107

**Published:** 2026-02-19

**Authors:** N. Ruswa, G. Günther, M. Claassens

**Affiliations:** ^1^ School of Medicine, Faculty of Health Sciences and Veterinary Medicine University of Namibia Windhoek Namibia; ^2^ National TB and Leprosy Programme, Directorate of Special Programmes Ministry of Health and Social Services Windhoek Namibia; ^3^ Department of Pulmonology, Allergology and Clinical Immunology, Inselspital, Bern University Hospital University of Bern Bern Switzerland

**Keywords:** HIV, isoniazid, preventive therapy, TPT, tuberculosis

## Abstract

**Background:**

Tuberculosis is a leading infectious cause of death globally and the main cause among those with HIV. TB preventive treatment (TPT) is key for preventing TB in people with HIV (PWH). Healthcare workers often worry about adverse effects of TPT regimens, including breakthrough TB–active TB during treatment. The burden of breakthrough TB remains largely unknown. This study aimed to determine the incidence and factors associated with breakthrough TB in PWH receiving HIV care.

**Methods:**

We analysed the nationwide electronic patient management system (EPMS) for HIV care and TB registers in Namibia, following PWH registered for HIV care to determine TB incidence before, during and after TPT and applying logistic regression to determine factors associated with breakthrough TB.

**Results:**

Of 334,606 PWH reviewed, 46,199 developed TB before, during, or after receiving TPT while being followed up for HIV care. PWH were followed for 114,667 patient‐years (PYs) while receiving TPT, with breakthrough TB incidence of 1.40 per 100 PYs (95% CI: 1.33–1.47). The 1790 true breakthrough cases had a median age of 37 (IQR 30–45). Median time to breakthrough TB was 2.5 months. Only 43 (2.4%) cases were drug‐resistant TB. In a logistic regression model comparing breakthrough versus non‐breakthrough TB cases, receiving ART increased breakthrough TB risk (aOR = 2.88; 95% CI: 1.07–7.78). The 3 month isoniazid‐rifapentine TPT regimen showed lower risk (aOR = 0.46; 95% CI: 0.36–0.63) than the 6 month isoniazid regimen, while 9H increased risk (aOR = 1.44; 95% CI: 1.18–2.52) but incidence rates were comparable between the regimens.

**Conclusion:**

The incidence of breakthrough TB was low at 1.4 per 100 PYs of TPT provision but indicating a need for improved screening of PWH before TPT. The incidence rate of TB among PWH during and after the shorter TPT regimen (3HP) is comparable to that of the 6H and 9H regimens.

## 1 Introduction

Tuberculosis (TB) remains a significant public health issue, with an annual incidence of 10.8 million and an estimated 1.25 million deaths in 2023 [1]. Namibia is classified among the countries with the highest TB incidence rate of 468 per 100,000 per year. Namibia also has a high HIV prevalence of 12.6% of the adult population [2]. TB preventive treatment (TPT) is widely recommended to prevent TB, particularly in people with HIV [3, 4]. TPT prevents the replication of *Mycobacterium tuberculosis* during latent infection [5].

Breakthrough TB occurs when a person develops active TB disease despite being on preventive therapy [6]. Additionally, suboptimal treatment for TB disease, such as with TPT regimens which inherently apply mono or dual therapy, is known to lead to drug resistance [7, 8]. Effectively screening for active TB before administering TPT would reduce the risk of missing active TB cases and subsequently reduce the chance of breakthrough TB and drug resistance. However, the evidence of TPT leading to drug resistance when applied in clinical trials or programmatic settings is not strong [9]. There are limited data on breakthrough TB cases and even more limited data on the relationship between breakthrough TB cases and drug resistance. To date, there has been no formal assessment of breakthrough TB cases in Namibia.

In this study, we aimed to determine the incidence of breakthrough TB in a national cohort of PLHIV receiving TPT, characterise the cases and explore the associated factors.

## 2 Materials and Methods

### 2.1 Setting

The Ministry of Health and Social Services in Namibia introduced a programme for comprehensive HIV care in 2002, subsequently enrolling PLHIV for routine follow‐up, antiretroviral therapy (ART) and cotrimoxazole as per the eligibility outlined in the national guidelines. Although TPT was initially offered to some patients according to international guidelines, it was formalised in national guidelines in 2005 [10]. Guidelines for managing PLHIV have evolved over the years, achieving universal antiretroviral use for all PLHIV (treat‐all) since 2016. In the national guidelines, all asymptomatic PLHIV were required to receive at least one course of isoniazid for 6–9 months, with the rifapentine‐isoniazid combination introduced in 2021. Only those who screened negative for cough, fever, weight loss, night sweats and lymph node enlargement were considered eligible if there were no further contraindications (liver disease, alcoholism, poor adherence) and had never received TPT before. There was no requirement to confirm TB infection (e.g., through a tuberculin skin test or interferon gamma release assay) prior to administering in local practice. TB was diagnosed according to the National Guidelines for the Management of TB [10, 11, 12], which have been aligned with WHO recommendations over the years. Cases included bacteriologically confirmed or clinically diagnosed pulmonary or extrapulmonary TB.

### 2.2 Study Design

This was a retrospective cohort analysis using secondary data from records of patients who were registered for comprehensive HIV care in the public health sector of Namibia since the inception of the HIV programme, focusing on those who developed TB.

### 2.3 Study Population

All individuals with HIV who had complete electronic records accessible at the central level were considered for analysis. Individuals who were not captured in the electronic patient management system (EPMS) and those who had TB before enrolment in HIV care were also excluded from the survival analysis.

### 2.4 Data Collection

A patient record list exported as comma‐separated value (CSV) spreadsheets from the HIV EPMS was linked to another exported from the electronic TB registers using probabilistic linkage with RegistryPlus LinkPlus (CDC, Atlanta, USA) with a match score set at 8.1. First names, surnames, dates of birth and sex were used as blocking variables, and the same variables plus district were used to compute the match score. Subsequent data merging and analysis were conducted using Stata version 18 (Statacorp, College Station, Texas, USA).

### 2.5 Study Variables

The main outcome of interest was the occurrence of active TB, as defined by notification in the TB registers and further classified according to when it was diagnosed relative to TPT administration. A TB case was defined as a notification in the national registry following a diagnosis consistent with the National Guidelines for the Management of TB, including pulmonary or extrapulmonary presentation, as well as those with or without bacteriological confirmation. Breakthrough TB cases were defined as TB cases registered after commencing TPT but before the expected date of TPT completion. All other TB cases were classified as non‐breakthrough TB. Comprehensive HIV care variables included demographic characteristics, such as date of birth, age, sex, region and district, as well as dates registered for HIV care, dates started on ART, dates started on TPT and WHO clinical stage at enrolment. TB‐related variables included date registered with TB, date of TB treatment initiation, classification of TB and treatment outcome. The follow‐up time was scaled to years, with person‐time events measured in person‐years.

## 3 Statistical Analysis

The primary software used for the analysis was Stata version 18 (StataCorp LLC, 2023). Descriptive statistics consisted of frequencies and percentages, as well as means with standard deviation and medians with interquartile ranges for continuous variables. For the TB incidence rate analysis, records were split into TPT‐unexposed (no TPT) and TPT‐exposed (TPT administered) groups. The latter was further split into pre‐TPT, during TPT, and post‐TPT. Occurrence of TB was included if it occurred at any point from enrolment in HIV care to the last visit date, with breakthrough TB being defined as TB that occurred any time after TPT was started but before the scheduled TPT completion date. Records were excluded from further survival analysis if the dates were inconsistent. The overall incidence rates of active TB were computed per 100 person‐years of follow‐up period. Furthermore, all TB cases were included in the descriptive analysis of the characteristics of cases arising during the course of HIV care. Chi‐square tests were used to determine significant factor variables (at the 5% significance level). Subsequently, TB cases were grouped into two categories: breakthrough cases, occurring during TPT, and non‐breakthrough cases, occurring at any other point during comprehensive HIV care. Logistic regression was used to explore the factors associated with breakthrough TB. Significance levels were tested at *p* < 0.05, and confidence intervals (CI) were set at 95%.

## 4 Results

### 4.1 Characteristics of Participants

Of the 334,606 records that could be accessed for people receiving care for HIV, 63,078 could be linked to a TB episode. Notably, 46,199 TB episodes could be traced to have occurred during comprehensive HIV care. Almost half (21,309; 46.1%) of the cases occurred in those who never received TPT, while 13,846 (30.0%) occurred pre‐TPT among those who went on to receive TPT and 9254 (20.0%) occurred post‐TPT. Only 1790 (3.9%) were breakthrough cases that occurred during the course of TPT. Just over half of the TB cases occurred in females, with the majority of cases occurring within the 25–34‐ and 35–44‐year age groups (27.1% and 35.3%, respectively). Most of the people with TB (34,191; 74.0%) received cotrimoxazole prophylaxis and 42,432 (91.8%) received ART. These characteristics are shown in (Table 1).

**TABLE 1:** Characteristics of participants.

	TPT status					
	Never TPT	Pre‐TPT	During TPT (Breakthrough TB)	Post‐TPT	Total	Test
*N*	21,309 (46.1%)	13,846 (30.0%)	1790 (3.9%)	9254 (20.0%)	46,199 (100.0%)	
Gender						
Female	10,427 (49.0%)	7222 (52.2%)	911 (50.9%)	5110 (55.2%)	23,670 (51.2%)	< 0.001
Male	10,872 (51.0%)	6622 (47.8%)	879 (49.1%)	4144 (44.8%)	22,517 (48.8%)	
TB age‐group						< 0.001
0–4	300 (1.5%)	248 (1.8%)	13 (0.7%)	18 (0.2%)	579 (1.3%)	
5–14	498 (2.4%)	523 (3.9%)	44 (2.5%)	150 (1.7%)	1215 (2.7%)	
15–24	1215 (5.9%)	756 (5.6%)	135 (7.7%)	475 (5.3%)	2581 (5.8%)	
25–34	5728 (27.8%)	3992 (29.7%)	533 (30.4%)	1870 (20.9%)	12,123 (27.1%)	
35–44	7015 (34.0%)	5047 (37.5%)	578 (33.0%)	3156 (35.2%)	15,796 (35.3%)	
45–54	3842 (18.6%)	2137 (15.9%)	306 (17.5%)	2108 (23.5%)	8393 (18.7%)	
55–64	1480 (7.2%)	578 (4.3%)	110 (6.3%)	838 (9.4%)	3006 (6.7%)	
65+	538 (2.6%)	167 (1.2%)	34 (1.9%)	342 (3.8%)	1079 (2.4%)	
CPT given						< 0.001
No	6904 (32.4%)	2712 (19.6%)	401 (22.4%)	1991 (21.5%)	12,008 (26.0%)	
Yes	14,405 (67.6%)	11,134 (80.4%)	1389 (77.6%)	7263 (78.5%)	34,191 (74.0%)	
ART given						
No	3191 (15.0%)	318 (2.3%)	67 (3.7%)	191 (2.1%)	3767 (8.2%)	< 0.001
Yes	18,118 (85.0%)	13,528 (97.7%)	1723 (96.3%)	9063 (97.9%)	42,432 (91.8%)	
Died						< 0.001
No	17,178 (80.6%)	13,182 (95.2%)	1672 (93.4%)	8177 (88.4%)	40,209 (87.0%)	
Yes	4131 (19.4%)	664 (4.8%)	118 (6.6%)	1077 (11.6%)	5990 (13.0%)	
TB site						
Both	3 (0.0%)	2 (0.0%)	0 (0.0%)	2 (0.0%)	7 (0.0%)	0.479
Extrapulmonary	3238 (18.1%)	2011 (18.6%)	253 (16.8%)	1391 (17.5%)	6893 (18.0%)	
Pulmonary	14,694 (81.9%)	8823 (81.4%)	1255 (83.2%)	6543 (82.4%)	31,315 (81.9%)	
Multi‐drug resistant TB						0.023
No	20,759 (97.4%)	13,548 (97.8%)	1747 (97.6%)	9001 (97.3%)	45,055 (97.5%)	
Yes	550 (2.6%)	298 (2.2%)	43 (2.4%)	253 (2.7%)	1144 (2.5%)	
TB treatment outcome						< 0.001
Success (cured or completed)	8090 (73.7%)	7070 (91.7%)	783 (86.6%)	3213 (83.3%)	19,156 (81.7%)	
Died	1981 (18.0%)	216 (2.8%)	69 (7.6%)	444 (11.5%)	2710 (11.6%)	
Lost to follow‐up	596 (5.4%)	230 (3.0%)	31 (3.4%)	102 (2.6%)	959 (4.1%)	
Failed	313 (2.9%)	197 (2.6%)	21 (2.3%)	98 (2.5%)	629 (2.7%)	
TPT regimen						< 0.001
6H	N/A	8390 (76.9%)	774 (43.2%)	4503 (48.7%)	13,667 (62.3%)	
9H	N/A	834 (7.6%)	126 (7.0%)	613 (6.6%)	1573 (7.2%)	
3HP	N/A	1659 (15.2%)	53 (3.0%)	197 (2.1%)	1909 (8.7%)	
Not documented	N/A	24 (0.2%)	837 (46.8%)	3941 (42.6%)	4802 (21.9%)	

Abbreviations: 3hp, 3 month weekly rifapentine and isoniazid; 6h/9h, 6 or 9 months of daily isoniazid; ART, antiretroviral therapy; CI, confidence interval; CPT, cotrimoxazole preventive therapy; OR, odds ratio; tpt, tb preventive therapy.

### 4.2 Characteristics of Patients With Breakthrough TB

Females had a slight predominance among the 1790 patients with breakthrough TB (911 females = 50.9%; 879 males). As with other forms of TB, the largest number of cases was observed in the 25–44‐year age group (63.4%). The majority (1389, 77.6%) had received cotrimoxazole, and almost all (1723, 96.3%) had received ART. Most (1255; 83.2%) had pulmonary TB, with 16.8% having extrapulmonary TB. Only 43 (2.4%) had drug‐resistant TB, and following treatment for TB, 86.6% had a successful treatment outcome. In comparison, the treatment success rates for the No TPT, Pre‐TPT and Post‐TPT groups were 73.7%, 91.7% and 83.3%, respectively. The majority of breakthrough cases were on the 6H regimen (774, 80.8%), while 126 (13.2%) were on the 9H regimen and 53 (5.5%) were on the 3HP regimen.

### 4.3 Incidence of Breakthrough TB

As shown in (Table 2), the overall incidence rate of TB for people receiving comprehensive HIV care was 1.96 per 100 PYs. Those who never had TPT had the highest incidence rate at 5.30 per 100 PYs (95% CI: 5.22–5.37), while the pre‐TPT group had a lower incidence rate at 2.12 per 100 PYs (95% CI: 2.08–2.15). The incidence rate of breakthrough TB (during TPT) was 1.40 per 100 (95% CI: 1.33–1.47). Post‐TPT, the incidence rate of TB was much lower at 0.85 (95% CI: 0.85–0.87).

**TABLE 2:** Incidence rate of TB by TPT status.

TPT status	Person‐years	Incidence rate (per 100 PYs)	95% CI
Never on TPT	347,502.8	5.30	5.22–5.37
Pre‐TPT	596,891.8	2.12	2.08–2.15
During TPT (breakthrough TB)	114,667.2	1.40	1.33–1.47
Post‐TPT	1,063,463.5	0.85	0.83–0.87
Total	2,122,525.2	1.96	1.94–1.98

The incidence rate of breakthrough TB (per 100 person‐years) by the regimen used is shown in (Table 3). The 6H and 9H regimens were associated with a similar incidence rate of breakthrough TB (1.13 and 1.07 per 100 PYs, respectively), while 3HP was associated with a higher incidence rate at 1.56 per 100 PYs (95% CI: 1.16–2.09) although the difference was not statistically significant. The incidence rate was the highest when the TPT regimen was not documented. Additionally, (Table 3) shows the post‐TPT incidence rate, demonstrating similar incidence rates across the 6H, 9H, and 3HP regimens. Those with undocumented regimens had a higher incidence rate at 1.32 cases per 100 PYs (95% CI: 1.28–1.37).

**TABLE 3:** Incidence rate of TB during and post TPT by regimen.

TPT regimen	Breakthrough TB (During TPT)		Post‐TPT TB (After completion)	
	IR (per 100 PYs)	95% CI	IR (per 100 PYs)	95% CI
6 Month isoniazid (6H)	1.13	1.05–1.22	0.68	0.66–0.70
9 Month isoniazid (9H)	1.07	0.89–1.29	0.66	0.61–0.72
3 Month rifapentine/isoniazid (3HP)	1.56	1.16–2.09	0.71	0.62–0.82
Not documented	1.92	1.79–2.07	1.32	1.28–1.37
Total	1.40	1.33–1.47	0.85	0.83–0.87

Abbreviations: 3HP, 3 month weekly rifapentine and isoniazid; 6H/9H, 6 or 9 months of daily isoniazid; CI, confidence interval; INH, isoniazid; IR, incidence rate; PYs, person‐years.

### 4.4 Timing of Breakthrough TB

The overall median time to breakthrough TB was 2.5 months (IQR 0.7–4.6), which varied by the regimen used: 2.5 for the 6H regimen (IQR 0.6–4.3), 1.7 months (IQR 0.5–2.9) for the 3HP regimen, and 3.7 months (IQR 1.6–6.9) for the 9H regimen.

### 4.5 Factors Associated With Breakthrough TB

A multivariate logistic regression model was fitted to include sex, age group, cotrimoxazole exposure, ART exposure and TPT regimen in predicting breakthrough TB cases (Table 4). Sex (OR = 1.07; 95% CI: 0.94–1.23), cotrimoxazole exposure (OR = 1.04; 95% CI: 0.88–1.23) and having multi‐drug‐resistant TB (OR = 0.99; 95% CI 0.64–1.53) were not significantly associated with being a breakthrough case. Comparing with the largest age group (35–44 years), being in the 15–24‐year age group was significantly associated with being a breakthrough case (OR = 1.37; 95% CI: 1.04–1.80). No other age groups showed a significant association. However, being on ART had almost three times the odds of being a breakthrough case (OR = 2.88; 95% CI: 1.07–7.78). Additionally, compared with the standard 6H regimen, being on the longer 9H regimen increased the odds of having breakthrough TB by 44% (OR = 1.44; 95% CI: 1.18–1.76), while being on the shorter 3HP regimen reduced the odds (OR = 0.47; 95% CI: 0.36–0.63). Having an undocumented regimen increased the odds, but with wide CI (OR = 1.47; 95% CI: 0.53–4.10).

**TABLE 4:** Logistic regression for factors associated with breakthrough TB.

Characteristic	Category	Odds ratio (OR)	95% CI	*p*
Sex	Ref (Female)	1.00		
	Male	1.07	0.94–1.23	0.295
Age group	Ref (35–44)	1.00		
	0–4	0.67	0.33–1.37	0.275
	5–14	0.89	0.59–1.35	0.589
	15–24	1.37	1.04–1.80	0.026
	25–34	1.13	0.95–1.33	0.170
	45–54	1.00	0.82–1.20	0.960
	55–64	1.05	0.78–1.41	0.746
	65+	0.80	0.46–1.39	0.428
CPT exposure	Ref (No)			
	Yes	1.04	0.88–1.23	0.656
ART exposure	Ref (No)	1.00		
	Yes	2.88	1.07–7.78	0.037
Multi‐drug resistant TB	Ref (No)	1.00		
	Yes	0.99	0.64–1.53	0.970
TPT regimen	Ref (6H)			
	9H	1.44	1.18–1.76	< 0.001
	3HP	0.47	0.36–0.63	< 0.001
	Not documented	1.47	0.53–4.10	0.461

Abbreviations: 3HP: 3‐month weekly rifapentine and isoniazid; 6H/9H: 6 or 9 months of daily isoniazid; ART: antiretroviral therapy; CI: confidence interval; CPT: cotrimoxazole preventive therapy; OR: odds ratio; TPT: TB preventive therapy.

## 5 Discussion

Studies on breakthrough TB are rare in the literature. In this first study of its kind in Namibia, we characterised 1790 cases of breakthrough TB in a cohort of over 300,000 PLHIV, of whom 215,815 received TPT. Breakthrough cases occurred at an incidence rate of 1.40 cases per 100 PYs (95% CI: 1.33–1.47). This was significantly lower than that in those who never received TPT (5.30 per 100 PYs; 95% CI: 5.22–5.37) and a third lower than that in the pre‐TPT period. However, this incidence rate was higher than that in the post‐TPT period. Although not conclusive, these findings support existing evidence demonstrating the association of TPT with a lower risk for TB. Akolo et al. [13] demonstrated that TPT reduced the risk of TB by 62% among those with TB infection and by 11% among those without TB infection. Later studies demonstrated the effectiveness of TPT, even among those taking ART [14], and its additive effect to that of ART. Both actively taking and having taken TPT were protective against TB. Two studies in South Africa and Botswana reported the efficacy of actively taking TPT in high‐transmission settings, which was lost after it was stopped [15, 16, 17]. Our study demonstrated similar post‐TPT incidence rates across the 6H, 9H and 3HP regimens, suggesting comparable efficacy. These findings support a longer durability of TPT than previously reported. This could be because transmission in our setting was not as high as in the settings of those studies. Supporting the possible underestimation of the durability of TPT in earlier studies, later studies did not demonstrate the benefits of repeated TPT courses [18]. In our study, having taken ART was associated with increased odds of having breakthrough TB. This may have been as a result of unmasking immune reconstitution inflammatory syndrome (IRIS) occurring soon after starting both ART and TPT [19]. Unmasking IRIS may also be responsible for the incidence rate of TB being apparently higher during compared to post‐TPT, with many PWH starting both TPT and ART shortly after enrolment as prescribed in the national guidelines [20], hence the higher risk.

Interestingly, the pre‐TPT period appeared to be protective against TB compared to never having received TPT, but this finding may be due to selection bias. Some earlier versions of national guidelines suggested that PLHIV who had already received TPT were no longer eligible for routine TPT [10], partly due to the reported protective efficacy of TB treatment itself. This may have resulted in clinicians generally not prioritizing those who had previously received treatment for TB for TPT; hence, those who never got TB would be selected for TPT and have a documented pre‐TPT period. Additionally, pre‐TPT screening would have weeded out those with symptoms associated with TB, making it less likely that those later offered TPT would have TB.

In a study by Yirdaw et al. [6], the incidence of breakthrough TB among PLHIV was 1106 per 100,000 PYs (95% CI: 742–1651), well within the rate that we found. In the study by Yirdaw, TB incidence after completing IPT was lower than the incidence of breakthrough TB at 624 per 100,000 PYs (95% CI: 488–797), consistent with our findings. We found that 30% of breakthrough TB cases were diagnosed within the first month of initiating TPT, similar to the 29% reported by Yirdaw et al. Despite these similarities, the study by Yirdaw had a much smaller sample of breakthrough TB cases [21] compared with our sample of 1790. In another study on breakthrough TB cases among PLHIV in Zambia, Nyangu et al. [22] reported an incidence of 267 per 100,000 PYs, with a sample of 130 cases, which was significantly lower than our finding equivalent to 1400 per 100,000 PYs. The Zambian study included patients enrolled from 2016 to 2019, in a treat‐all era [23], where all PLHIV were offered early ART and TB incidence rates were much lower. The Ethiopian cohort reported by Yirdaw was between 2005 and 2013, when incidence rates were higher, and ART access was reserved for PLHIV with advanced disease. In a clinical trial in Botswana comparing patients on a 6 month versus a 36 month isoniazid TPT, the incidence of active TB was 1.3 per 100 PYs and 1.1 per 100 PYs, respectively, and 47% of the patients had access to ART at the end of their TPT [24]. These findings are comparable to our own.

Our study included data from 2002, when the TB incidence rates were significantly higher.

In an Indian cohort of 1492 PLHV who started TPT, only four cases of breakthrough TB were noted, accounting for 2% of the reasons for stopping TPT [21]. Incidentally, a modelling study on the cost‐effectiveness of TPT among PLHIV in South Africa predicted that, at a 7.9% reactivation rate, offering 6 months of isoniazid preventive therapy to 100,000 PLHIV would result in 1400 breakthrough cases in the first year [25], which is similar to our finding. That study used assumptions derived from Narain et al. [26], who estimated the incidence of HIV‐associated TB to be 5%–8% among PLHIV in high‐burden settings. Our finding of 5.3 cases per 100 PYs among those who never received TPT also falls within this range. In a large cohort of gold miners in South Africa [27], the incidence of TB during intended TPT administration was 1.3 per 100 PYs, similar to our finding. However, when the actual TPT duration and adherence were considered, the incidence decreased. In our analysis, the actual duration and adherence data were not available, but our cohort may be representative of the heterogeneity expected in real‐world populations.

One limitation of this study was its retrospective design, which restricted the range of variables available for the analysis. Furthermore, the analysis depended on programmatic data that were routinely collected and later entered into electronic medical records retrospectively. This method of data entry is prone to missing details, inaccuracies and misclassification, potentially introducing bias in the determination of exposures and outcomes. The large cohort size and extended follow‐up period enhanced the robustness of the results. To define TB cases, programmatic definitions were used without documenting bacteriological confirmation. Consequently, clinically diagnosed cases were included alongside bacteriologically confirmed cases, but there were no data to distinguish the method of confirmation, opening the possibility of misdiagnoses. However, the size of the cohort and the fact that national guidelines were used, which align with World Health Organization guidelines, may have minimized the effect of misdiagnoses. Another limitation was the unavailability of data on ART start dates relative to TPT start dates, which precluded further analysis of the effect of ART timing on the incidence rates of breakthrough TB.

## 6 Conclusion

These findings confirm the occurrence of TB cases even among those who are on TPT, albeit at much lower incidence rates than for those who never received TPT. While the rates of breakthrough TB are not alarming compared to elsewhere, there is a need to strengthen TB screening prior to starting TPT and during TPT. The incidence rates of TB during and post‐TPT are comparable across the 6–9 month isoniazid regimens and the 3HP regimen.

## Ethics Statement

Ethical Approval was obtained from the University of Namibia Research Ethics Committee (reference number SOM06/2023) and permission was granted by the Namibia Ministry of Health and Social Services (MoHSS). Informed consent was waived because this was a secondary analysis of data with minimal direct consequences for the participants. Although names and other identifiers were used in the data linkage process, they were subsequently deleted and not used in the dataset used in the present analysis.

## Conflicts of Interest

The authors declare no conflicts of interest.

## Data Availability

The data that support the findings of this study are available on request from the corresponding author. The data are not publicly available due to privacy or ethical restrictions.
